# Pembrolizumab Activity in Tyrosine Kinase Inhibitor–Refractory Metastatic GIST: A Case Report

**DOI:** 10.1002/cnr2.70726

**Published:** 2026-11-04

**Authors:** Neharika Bandaru, Job Shiach, Mark Agulnik

**Affiliations:** ^1^ USC Norris Comprehensive Cancer Center Keck School of Medicine of the University of Southern California Los Angeles California USA; ^2^ Department of Internal Medicine Keck School of Medicine of the University of Southern California Los Angeles California USA; ^3^ Los Angeles General Medical Center Los Angeles California USA

## Abstract

**Introduction:**

Gastrointestinal stromal tumors (GISTs) are commonly driven by activating mutations in *KIT* or *PDGFRA*. Although sequential tyrosine kinase inhibitors (TKIs) have improved outcomes in advanced disease, acquired resistance is common. Prospective studies of immune checkpoint blockade in GIST have shown limited activity, and immunotherapy is not a standard treatment.

**Case Presentation:**

A man diagnosed at age 57 with epithelioid small‐bowel GIST developed hepatic metastatic disease 7 years after his initial diagnosis. His tumor harbored a primary *KIT* exon 11 D579del mutation, a secondary *KIT* exon 17 D820G mutation, and *CDKN2A/CDKN2B* deletions; available molecular testing reported a low tumor mutational burden (1 mutation/Mb) and microsatellite‐stable disease. After progression or treatment‐limiting toxicity during six TKI regimens, pembrolizumab was initiated on October 28, 2025, when he was 76 years old, and administered intravenously every 3 weeks. The treatment has demonstrated stable disease on CT imaging; cycle 13 was then administered on July 28, 2026. No treatment delays or treatment‐related adverse events were reported through cycle 13.

**Conclusion:**

Pembrolizumab was associated with at least 9 months of disease control in TMB‐low, microsatellite‐stable metastatic GIST after six TKI regimens. Because contemporaneous immune profiling and complete historical dosing data were unavailable, this observation is hypothesis‐generating and does not establish that prior TKI exposure sensitized the tumor to PD‐1 blockade.

## 1 Introduction

Gastrointestinal stromal tumors (GISTs) are the most common mesenchymal neoplasms of the gastrointestinal tract, with an estimated annual incidence of approximately 10–15 cases per million people [1]. They are thought to arise from interstitial cells of Cajal or related precursor cells and are commonly driven by activating mutations in *KIT* or platelet‐derived growth factor receptor alpha (*PDGFRA*) [2].

Imatinib substantially improved outcomes for patients with metastatic GIST [3]. Nevertheless, most patients with advanced disease eventually develop resistance, frequently through secondary *KIT* mutations, including activation‐loop mutations in exon 17 [4]. Sunitinib, regorafenib, and ripretinib provide additional disease control after imatinib failure, but resistance or intolerance ultimately limits these therapies [5, 6, 7].

Immune checkpoint blockade has not demonstrated sufficient activity to become a standard treatment for GIST. In a randomized Phase II study, neither nivolumab monotherapy nor nivolumab plus ipilimumab met the prespecified response‐rate benchmark [8]. Current reviews emphasize that single‐agent PD‐1 blockade has generally underperformed in GIST despite biologic evidence of immune infiltration and multiple immunosuppressive or exhaustion pathways [9].

We report sustained disease control with pembrolizumab in a patient with TMB‐low, microsatellite‐stable metastatic small‐bowel GIST after progression or intolerance during six TKI regimens. The distinctive feature of this case is durable stable disease with heterogeneous lesion‐level improvement in an ultra‐heavily pretreated setting in which prospective checkpoint‐inhibitor studies have shown limited activity.

## 2 Case Report

In 2007, a 57‐year‐old man with a history of prostate cancer treated with prostatectomy was diagnosed with epithelioid small‐bowel GIST. He underwent surgical resection and received 12 months of adjuvant imatinib in line with ACOSOG Z9001 [10]. Surveillance imaging in 2014, 7 years after the initial diagnosis, demonstrated metastatic disease involving the liver.

Imatinib was restarted, and the patient initially achieved disease control. Two resistant hepatic lesions subsequently grew and were surgically resected. Pathologic examination demonstrated high‐grade epithelioid GIST with positive CD117 and DOG‐1 staining. Genomic analysis identified a primary *KIT* exon 11 D579del mutation, a secondary *KIT* exon 17 D820G mutation, and deletions of *CDKN2A* and *CDKN2B*. GEM ExTra report from liver lesion excised December 2019 documented low tumor mutational burden (1 mutation/Mb) and microsatellite‐stable disease.

The treatment course is summarized in Table 1 and Figure 1. Regorafenib was trialed in 2019 on the basis of the sequencing report but was poorly tolerated. Ripretinib was initiated in 2020 and maintained disease control until August 2024, when progression prompted dose escalation from 150 mg once daily to 150 mg twice daily and Y‐90 radioembolization of enlarging hepatic lesions. Ripretinib was discontinued in October 2024 after progression with multiple new hepatic lesions, peritoneal soft‐tissue nodules, pulmonary micronodules, and mesenteric nodules, accompanied by worsening ascites and a right pleural effusion.

**TABLE 1:** Systemic and liver‐directed therapies for this patient with metastatic GIST, with dates, dosing, best response, and reason for discontinuation.

Therapy/intervention	Dates	Dose and schedule	Best outcome	Reason for discontinuation/status
Primary resection	2007	Not applicable	Resection of primary small‐bowel GIST	Not applicable
Adjuvant imatinib	2007–2008 (12 months)	400 mg daily	Seven years from diagnosis to metastatic recurrence	Planned completion; patient was not enrolled in Z9001
Imatinib for metastatic disease	2014–2019	400 mg daily	Initial control; two resistant hepatic lesions resected	Focal hepatic progression/transition to later therapy
Regorafenib	2019—Oct 2020	160 mg daily for 21 days on, 7 days off	NR	Poor tolerability; anemia, reduced renal function
Ripretinib	2020–Oct 2024	150 mg daily until Aug 2024 when dose increased to BID due to progression.	Stable disease until dose escalation. Y‐90 targeted enlarging hepatic lesions	Multisite progression
Sunitinib	Dec 2024; several weeks	37.5 mg daily, decreased to 25 mg daily	Short exposure	Anemia, thrombocytopenia, fatigue
Cabozantinib	Jan–May 2025	40 mg daily	Progressive disease	Radiographic progression
Avapritinib	May–Jun 2025	300 mg daily	Short exposure	Cognitive impairment; body shaking, dizziness
Imatinib rechallenge	Jun–Aug 2025	400 mg daily	Progressive disease	Radiographic progression
Pembrolizumab	Oct 28, 2025–Jul 28, 2026; ongoing at follow‐up	200 mg IV every 3 weeks	Stable disease through cycle 13	Ongoing; no delays or adverse events reported

Abbreviations: NR, not reported in available records; Y‐90, yttrium‐90.

**FIGURE 1:**
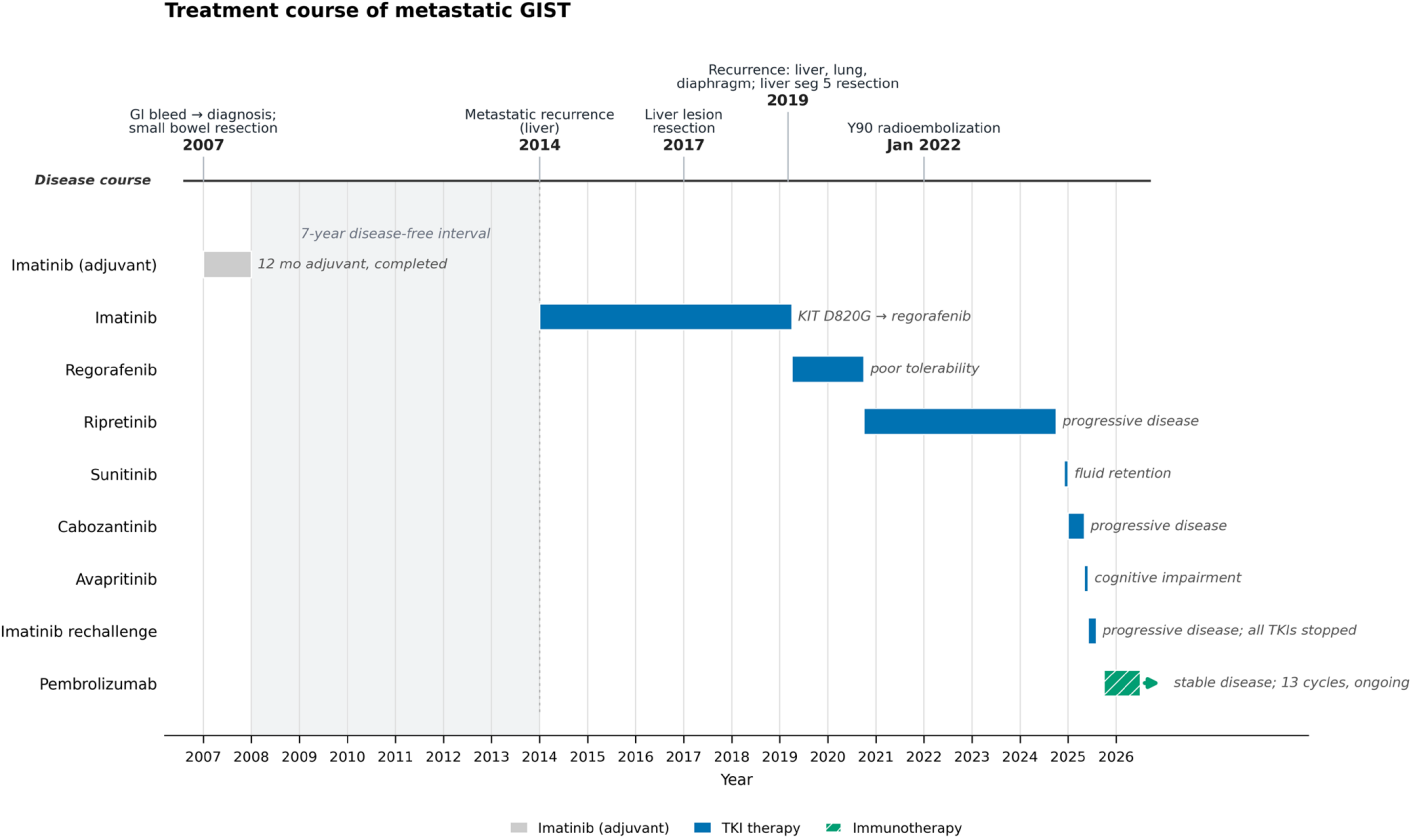
Treatment and disease‐course timeline for metastatic small‐bowel GIST. The timeline summarizes the initial resection in 2007, 12 months of adjuvant imatinib, hepatic metastatic recurrence in 2014, subsequent systemic and liver‐directed therapies, and pembrolizumab treatment through cycle 13 on July 28, 2026. Ripretinib began in 2020, maintained disease stability until August 2024, was later escalated to twice‐daily dosing, and was discontinued in October 2024. Pembrolizumab began on October 28, 2025; four cycles preceded the January 2026 scan, eight preceded the April 20, 2026 scan, and cycle 13 followed CT imaging on July 23, 2026. Abbreviations: GIST, gastrointestinal stromal tumor; TKI, tyrosine kinase inhibitor; Y‐90, yttrium‐90.

Sunitinib was initiated in December 2024 but was discontinued within weeks because of significant fluid retention. Cabozantinib was administered from January through May 2025 and discontinued after CT demonstrated progression with multifocal hepatic metastases, extensive peritoneal disease, and retroperitoneal disease. The largest hepatic lesion, located in segment IVB, measured 5.7 cm. Avapritinib was administered from May through June 2025 and stopped because of cognitive impairment and dizziness. Imatinib was restarted in June 2025, but subsequent imaging demonstrated continued progression of abdominal, pelvic, and suspected pulmonary metastases with persistent ascites and pleural effusion. All TKI therapy was discontinued in August 2025.

MRI of the abdomen and pelvis on August 19, 2025, obtained during imatinib re‐challenge, demonstrated progression of intra‐abdominal and pelvic metastatic disease. Findings included enlargement of preexisting peritoneal lesions together with new peritoneal metastases, increasing hepatic and perihepatic metastases, increasing mild intra‐abdominal ascites, and an increasing moderate right pleural effusion. A stable 5 mm pleural‐based enhancing nodule in the medial right lower lobe was considered suspicious for metastasis. All TKI therapy was discontinued shortly thereafter, and pembrolizumab was initiated 70 days later.

Throughout this patient's time on TKIs, he struggled with poor renal function and anemia, requiring multiple hospitalizations for melena and hematochezia. Hemoglobin ranged from 6.3 to 10.5 g/dL, and creatinine averaged approximately 1.5 mg/dL, corresponding to an estimated glomerular filtration rate of approximately 45 mL/min/1.73 m^2^.

Overall, the patient had received six TKIs across seven treatment courses: imatinib, regorafenib, ripretinib, sunitinib, cabozantinib, and avapritinib, with imatinib subsequently used as a rechallenge. This included all TKIs approved for KIT‐mutant GIST as well as two agents used off‐label.

After this progression, the patient was screened for the Phase 1a/1b KOMET‐015 study of ziftomenib, a menin inhibitor, in combination with imatinib [11], based on preclinical evidence that menin inhibition reduces GIST growth and enhances imatinib activity in patient‐derived xenograft models [12]. He was ineligible because of anemia and progressive renal dysfunction. His performance status did not preclude additional therapy (ECOG 1). Repeat locoregional therapy was not considered appropriate given the predominantly peritoneal distribution of progression. With no remaining standard or accessible investigational options, the alternatives were transition to hospice care or an empiric trial of therapy outside the KIT‐directed class, and off‐label pembrolizumab was elected after discussion of its experimental status in GIST and its comparatively favorable toxicity profile.

Pembrolizumab was initiated on October 28, 2025, following a 70‐day interval without systemic therapy, and administered at 200 mg intravenously every 3 weeks. The patient has completed 13 cycles at the time of this writing with continued stable disease, as shown in Table 2. Response was assessed retrospectively by the authors according to RECIST 1.1 [13], as the original radiology reports did not include formal response assessment. Because pretreatment imaging was performed without intravenous contrast owing to renal impairment, the contrast‐enhanced CT of January 2026 served as the measurement baseline. Two peritoneal implants designated as index lesions on that study—one along the serosal surface of hepatic segments 4/5 and one peripancreatic—were followed as target lesions; remaining peritoneal deposits and the 3–4 mm pulmonary micronodules were classified as nontarget. The sum of target lesion diameters decreased from 9.9 cm at baseline to 9.4 cm in April 2026 and 8.0 cm in July 2026, a 19.2% reduction (Figures 2 and 3). With no new lesions and nontarget disease stable to slightly decreased, best overall response was stable disease. Comparison with the October 2025 PET/CT, on which the dominant implant measured 11.0 cm, suggests a substantially larger reduction, but spans a change in contrast technique and is reported as exploratory.

**TABLE 2:** Pembrolizumab administration and radiographic assessments by study day. Day 0 is the first infusion; imaging studies are shaded, and pre‐treatment studies appear above the rule.

Study day	Date	Intervention/assessment	Findings
−70	Aug 2025	MRI abdomen and pelvis (during imatinib rechallenge)	Progression of intra‐abdominal and pelvic metastatic disease: enlargement of preexisting peritoneal lesions with new peritoneal metastases, increasing hepatic and perihepatic metastases, increasing mild ascites, and increasing moderate right pleural effusion. Stable 5 mm pleural‐based enhancing nodule in the medial right lower lobe, suspicious for metastasis.
−18	Oct 2025	PET/CT (non‐contrast)	New and increasing peritoneal deposits compared with March 2025: bladder dome 1.1 → 3.9 cm (SUV 7.5), right lower peritoneal 1.5 → 4.7 cm (SUV 8.9), right mesenteric 2.1 → 4.0 cm (SUV 12), peripancreatic mesenteric 3.6 → 4.0 cm, plus new 1.9 cm left lower mesenteric (SUV 7.1) and 1.3 cm left peritoneal deposits. Increasing hepatic metastases: segment 4B/5 lesion 7.4 → 11.0 cm (SUV 12) with central necrosis; segment 4A lesion 6.1 → 6.5 cm (SUV 13). Calcified and fluid‐attenuation hepatic lesions consistent with prior Y‐90 effect. Likely stable 1.5 cm right middle lobe subpleural nodule, suspicious for metastasis; minimally increased bilateral pulmonary solid nodules. Small right and trace left pleural effusions. Nonspecific esophageal dilatation.
0	Oct 2025	Pembrolizumab, cycle 1	—
21	Nov 2025	Pembrolizumab, cycle 2	—
42	Dec 2025	Pembrolizumab, cycle 3	—
63	Dec 2025	Pembrolizumab, cycle 4	—
84	Jan 2026	CT chest with contrast	Interval decrease in size of previously noted micronodules in the right and left lung (all 3–4 mm); follow‐up recommended to confirm complete resolution. Interval decrease in right pleural fluid, small on the current examination. Multifocal pleuroparenchymal scarring with subsegmental atelectasis and clusters of tiny micronodules, favored to represent sequelae of prior processes such as recurrent aspiration. Esophageal abnormality noted.
86	Jan 2026	CT abdomen and pelvis with contrast	Numerous peritoneal tumor implants, decreased in size compared with the October 2025 PET/CT, allowing for differences in technique. Served as the baseline for RECIST 1.1 measurement.
91	Jan 2026	Pembrolizumab, cycle 5	—
112	Feb 2026	Pembrolizumab, cycle 6	—
133	Mar 2026	Pembrolizumab, cycle 7	—
154	Mar 2026	Pembrolizumab, cycle 8	—
174	Apr 2026	CT chest and CT abdomen and pelvis with contrast	Abdomen and pelvis: stable to slightly decreased size of numerous low‐attenuation peritoneal tumor implants since January 2026. Chest: stable clustered bibasilar micronodules, likely sequelae of prior infectious or inflammatory process including aspiration; a few additional stable indeterminate pulmonary micronodules; diffusely patulous esophagus; no enlarged intrathoracic lymph nodes; stable small right pleural effusion.
182	Apr 2026	Pembrolizumab, cycle 9	—
203	May 2026	Pembrolizumab, cycle 10	—
219	Jun 2026	Pembrolizumab, cycle 11	—
252	Jul 2026	Pembrolizumab, cycle 12	—
268	Jul 2026	CT chest and CT abdomen and pelvis with contrast	Abdomen and pelvis: stable to slightly decreased size of numerous low‐attenuation peritoneal tumor implants since April 2026; ascites no longer reported; increased distention of the rectum with stool. Chest: stable pulmonary micronodules with no new nodules; stable small right pleural effusion; centrilobular tree‐in‐bud nodularity involving the lingula and right lower lobe, likely aspiration‐associated given the esophageal findings; patulous esophagus with circumferential distal wall thickening, which may reflect esophagitis, with endoscopic evaluation suggested if clinically indicated.
273	Jul 2026	Pembrolizumab, cycle 13	—

*Note:* Study Day 0 denotes the first pembrolizumab infusion; negative values precede it. Shaded rows indicate imaging assessments; the two rows above the rule are pretreatment studies. Pretreatment imaging was performed without intravenous contrast or as MRI because of renal dysfunction; contrast‐enhanced CT became feasible after renal recovery, and measurements were therefore referenced to the January 2026 contrast‐enhanced study rather than to a pretreatment baseline. Treatment was administered on a 21‐day cycle except between Cycles 10 and 11 (16 days) and between Cycles 11 and 12 (33 days).

Abbreviations: CT, computed tomography; MRI, magnetic resonance imaging; PET, positron emission tomography; RECIST, response evaluation criteria in solid tumors.

**FIGURE 2:**
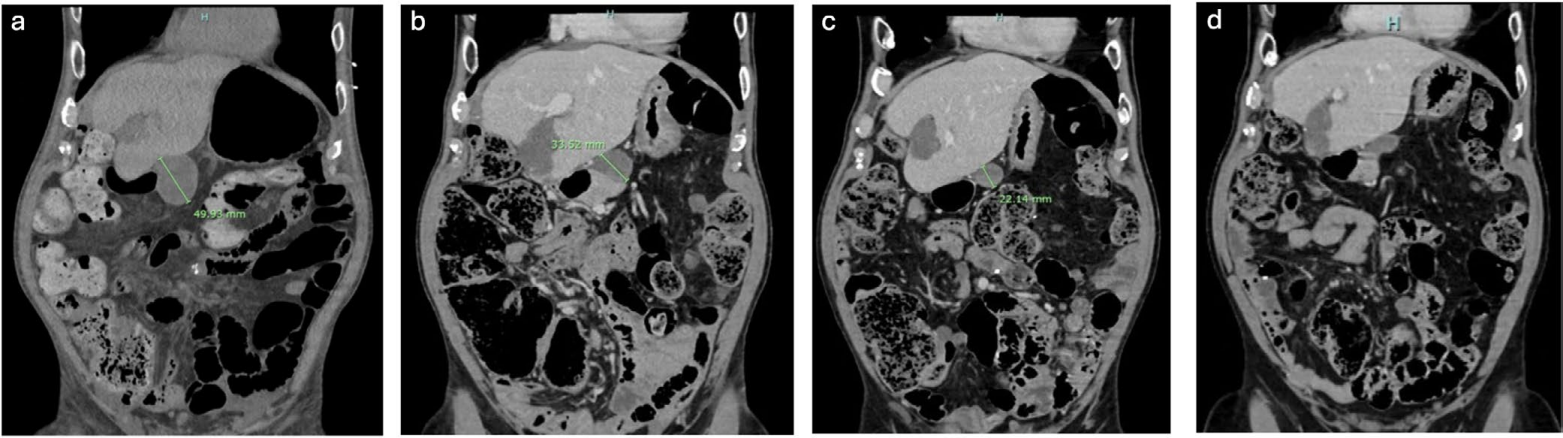
Coronal CT abdomen and pelvis (CTAP) showing radiographic stable disease of a large sub‐hepatic lesion to pembrolizumab. (a) March 2025, non‐contrast CTAP (performed without contrast due to reduced renal function). (b) January 2026, contrast‐enhanced CTAP after 3 months of pembrolizumab. (c) April 2026, contrast‐enhanced CTAP after 8 cycles of pembrolizumab. (d) July 2026, contrast‐enhanced CT abdomen and pelvis.

**FIGURE 3:**
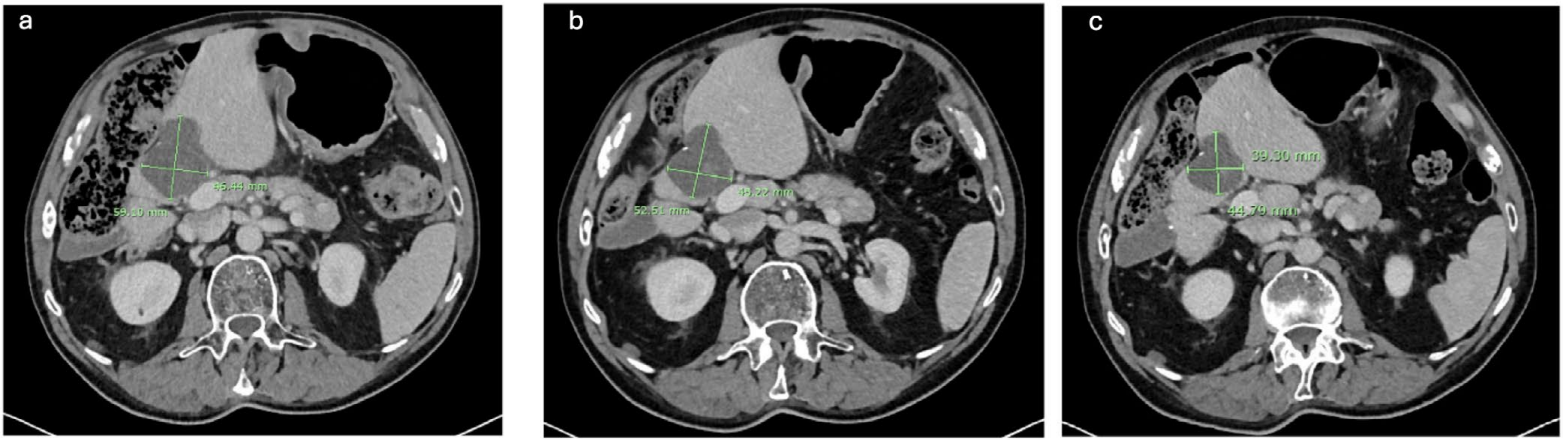
Axial contrast‐enhanced CT abdomen and pelvis of the largest hepatic lesion in segment IVB at (a) January 2026, (b) April 2026, and (c) July 2026 demonstrating 7 months of stability with interval reduction in lesion size while on pembrolizumab monotherapy.

Creatinine was 1.58 mg/dL at imatinib discontinuation and had improved to 1.34 mg/dL by early October 2025, before pembrolizumab exposure, falling further to 1.24 mg/dL in February 2026 and 1.16 mg/dL in July 2026; this trajectory began during the treatment‐free interval and is most consistent with recovery from tyrosine kinase inhibitor–associated nephrotoxicity. Hemoglobin was 8.4 g/dL at imatinib discontinuation and remained low through that same interval (9.0 g/dL in early October 2025), rising only after pembrolizumab was started, to 13.1 g/dL by February 2026 and 12.6 g/dL in July 2026. Resolution of gastrointestinal blood loss, previously attributed to both tumor‐related bleeding and antiangiogenic therapy, is the most likely explanation. Through cycle 13 the patient experienced no documented treatment‐related or immune‐related adverse events, no dose interruptions due to adverse events (two doses were adjusted for scheduling: cycle 10 and 11), and no treatment discontinuation. He reported no fevers, chills, sweats, hemoptysis, or bowel or bladder dysfunction.

## 3 Discussion

This case describes 9 months of stable disease with pembrolizumab in a patient with advanced GIST who had progressed through or been unable to tolerate six TKI regimens. Serial CT demonstrated stable disease, with a decrease in a target hepatic lesion, a larger decrease in a separate subhepatic lesion, and continued stability of pulmonary and peritoneal disease through July 2026. In a patient this heavily pretreated, disease control of this duration is clinically meaningful. Equally notable is the improvement in the patient's long standing anemia and kidney function.

At the time of this decision, the patient had progressed through or been unable to tolerate 6 tyrosine kinase inhibitors (TKIs) for advanced GIST. He was screened for the Phase 1a/1b KOMET‐015 study of ziftomenib in combination with imatinib (NCT06655246) and was ineligible because of anemia and renal dysfunction. Although other investigational KIT inhibitors and combination regimens have demonstrated early clinical activity in refractory disease [14], the patient's cumulative intolerance across this class made further KIT‐directed therapy unlikely to be feasible. In the absence of remaining standard or accessible investigational options, pembrolizumab was offered as an empiric trial, selected principally for its more favorable toxicity profile rather than for established activity in GIST.

This outcome contrasts with the generally limited activity observed in prospective GIST immunotherapy studies. In the randomized Phase II study by Singh et al., the nivolumab arm had no objective responses, although 10 of 19 patients achieved stable disease; the nivolumab‐plus‐ipilimumab arm included one complete response among 16 patients and four patients with stable disease. Neither arm met the prespecified objective‐response benchmark [8]. Pembrolizumab has also not been prospectively established as an effective treatment for GIST [9], and its use in this patient was off‐label and empiric. The durable stable disease observed here resembles the stable‐disease signal reported in those trials rather than an objective response, and should be interpreted accordingly.

A striking feature of this case is that disease control occurred in a tumor lacking any established predictor of checkpoint inhibitor benefit. The tumor was TMB‐low at 1 mutation/Mb and microsatellite stable, and large molecular series indicate that TMB‐high and MSI‐high disease are uncommon in GIST [15]. PD‐L1 immunohistochemistry was not available in the records accessible to the authors and has been requested from the outside institution where the specimen was processed; no CD8 quantification, contemporaneous immune profiling, or repeat tissue or plasma sampling was obtained before initiation. Sequencing also identified CDKN2A/CDKN2B deletions, which have been associated with malignant progression in KIT‐mutant GIST [16] and are consistent with the observed disease course, in which pretreatment imaging documented near‐tripling of several peritoneal deposits and enlargement of the dominant hepatic lesion from 7.4 to 11.0 cm over 7 months. These alterations are not established predictors of checkpoint inhibitor response.

One hypothesis for why pembrolizumab resulted in disease stabilization in this patient is that sequential TKI exposure altered tumor biology or the immune microenvironment. Imatinib can potentiate antitumor T‐cell responses through inhibition of indoleamine 2,3‐dioxygenase in preclinical GIST models [17], and combined PD‐1/PD‐L1 blockade has enhanced imatinib activity in translational studies [18]. Yoo et al. found an association between exposure to multiple antiangiogenic TKIs and higher expression of PD‐1/PD‐L1 and other exhaustion markers in advanced GIST specimens [19]. However, that series also demonstrated a predominantly immunosuppressive phenotype and could not separate treatment effects from natural disease evolution. Without paired tissue or blood‐based immune profiling from the time of treatment, this case cannot establish whether prior TKI exposure sensitized the tumor to pembrolizumab.

Alternative explanations warrant consideration. Prior Y‐90 radioembolization could have contributed to a change in a previously treated hepatic lesion if that lesion lay within the treatment field, but this does not account for the concurrent stability of disease outside any prior radiation field, including peritoneal and pulmonary sites. Indolent natural history is difficult to reconcile with the August 2025 MRI, which documented enlarging peritoneal lesions, new peritoneal metastases, increasing hepatic disease, and increasing ascites and pleural effusion 10 weeks before initiation. Delayed benefit from prior TKI therapy is likewise unlikely, as that progression occurred during active imatinib exposure.

This single case cannot establish causation. Immune characterization was limited to archival TMB‐low, microsatellite‐stable status with no contemporaneous PD‐L1, CD8, or blood‐based profiling, so the immune context at initiation of pembrolizumab is unknown. Imaging assessment was retrospective, and the transition from non‐contrast to contrast‐enhanced technique after renal recovery precludes technique‐matched measurement across the treatment boundary; the lesion‐level comparison spanning that transition is exploratory. These constraints limit mechanistic inference but not the primary observation: disease progressing through TKI exposure stabilized on pembrolizumab for 9 months, ongoing at time of writing.

## 4 Conclusion

In summary, this case documents sustained and well‐tolerated disease control with pembrolizumab in a patient with GIST refractory to six lines of KIT‐directed therapy, in the absence of TMB‐high or MSI‐high status or any other established predictor of checkpoint inhibitor benefit. The observation is hypothesis‐generating rather than practice‐changing. It does, however, support prospective evaluation of checkpoint blockade in heavily pretreated GIST, ideally incorporating paired pre‐ and on‐treatment tissue to characterize the immune microenvironment in patients with prolonged TKI exposure.

## Author Contributions

**Neharika Bandaru:** writing – original draft, writing – review and editing, investigation. **Job Shiach:** writing – original draft, writing – review and editing, resources, project administration, visualization, methodology, investigation. **Mark Agulnik:** supervision, writing – review and editing, conceptualization, investigation.

## Funding

The authors have nothing to report.

## Ethics Statement

Institutional review board approval was not required for this single‐patient case report in accordance with institutional policy.

## Consent

Written informed consent was obtained from the patient for publication of the clinical details and accompanying images.

## Conflicts of Interest

The authors declare no conflicts of interest.

## Data Availability

Additional de‐identified information may be available from the corresponding author upon reasonable request, subject to patient privacy and institutional restrictions.
